# Exploring the efficacy of an electronic symptom assessment and self-care intervention to preserve physical function in individuals receiving neurotoxic chemotherapy

**DOI:** 10.1186/s12885-018-5093-z

**Published:** 2018-12-04

**Authors:** Robert Knoerl, Edie Weller, Barbara Halpenny, Donna Berry

**Affiliations:** 1 Phyllis F. Cantor Center for Research in Nursing and Patient Care Services, Dana-Farber Cancer Institute, 450 Brookline Avenue, LW 517, Boston, MA 02215 USA; 2 Biostatistics and Research Design Core, Institutional Centers for Clinical and Translational Research, Boston Children’s Hospital, 21 Autumn Street Suite 313, Boston, MA 02215 USA; 3 Phyllis F. Cantor Center for Research in Nursing and Patient Care Services, Dana-Farber Cancer Institute, 450 Brookline Avenue, LW 521, Boston, MA 02215 USA; 4 Phyllis F. Cantor Center for Research in Nursing and Patient Care Services, Dana-Farber Cancer Institute, 450 Brookline Avenue, LW 518, Boston, MA 02215 USA

**Keywords:** Peripheral nervous system diseases/chemically-induced, Chemotherapy-induced peripheral neuropathy, Electronic symptom assessment, Technology assessment, Cancer symptom management

## Abstract

**Background:**

Impaired physical function due to chemotherapy-induced peripheral neuropathy (CIPN) symptoms may lead to diminished quality of life. However, even with the knowledge of the effects of CIPN on physical function, clinicians infrequently assess and manage CIPN. Interventions that prioritize the early identification of CIPN to provide prompt treatment may reduce the impact of CIPN on physical function. The purpose of this paper is to compare self-reported physical function in individuals receiving neurotoxic chemotherapy between Electronic Symptom Assessment-Cancer (ESRA-C) intervention group (e.g., opportunity for symptom screening, self-care recommendations, communication coaching, and symptom tracking) and control group participants (i.e., electronic assessment alone). Secondary outcomes include pain intensity, sensory/motor CIPN, depression, fatigue, and insomnia.

**Methods:**

The data used in this paper are a subset of a randomized controlled trial that examined the impact of the ESRA-C intervention on symptom distress in individuals receiving cancer treatment. Since the interest in this analysis is on the effects of neurotoxic chemotherapy on physical function, subjects were included if they received platinum and/or taxane-based chemotherapy and completed the baseline and end-of-treatment measures. Participants completed standardized questionnaires of physical function, CIPN, fatigue, depression, pain intensity, and insomnia prior to treatment, 3–6 weeks after treatment initiation, and after the completion of treatment. Changes in mean scores are compared between groups using linear mixed models adjusting for age.

**Results:**

Intervention group participants reported significantly less reduction in physical functioning (baseline: 87.4/100; end-of-treatment: 84.5/100) relative to the control (baseline: 90.2/100; end-of-treatment: 81.8/100) (*p* = 0.011). For secondary measures, significantly less depression (*p* = 0.005) was observed in the intervention group as compared to the control, but otherwise, there were no between-group differences. Among participants who received high cumulative doses of neurotoxic chemotherapy, the intervention group reported significantly less severe sensory (*p* = 0.007) and motor CIPN (*p* = 0.039) relative to the control.

**Conclusion:**

Use of the ESRA-C intervention led to less reduction in physical function in comparison to the control in individuals receiving neurotoxic chemotherapy. Further research is needed to confirm our findings and to identify how electronic symptom assessment technology may mediate physical function preservation.

**Trial registration:**

ClinicalTrials.Gov NCT00852852. Registered 27 February 2009.

## Background

Approximately 4600 individuals are diagnosed with new hematological and/or oncological malignancies each day [1], of which many will require neurotoxic chemotherapy (e.g., platinums or taxanes) for the treatment of the cancer [2]. Chemotherapy-induced peripheral neuropathy (CIPN) occurs in up to 68% of individuals receiving neurotoxic chemotherapy [3] and results from chemotherapy-induced damage to peripheral nerves (e.g., “dying back” axon degeneration) [4]. Patients with CIPN symptoms may experience numbness, tingling, pain (sensory symptoms), or reductions in strength (motor symptoms) that present in a stocking-glove distribution [2]. CIPN may linger long after treatment completion if not adequately assessed and managed during neurotoxic chemotherapy treatment [5].

Several recent studies have investigated the impact of CIPN symptoms on physical function during and after neurotoxic chemotherapy treatment [6–9]. Overall, the data suggest that individuals with sensory and/or motor CIPN symptoms report a decrease in physical function and an increase in the number of falls in comparison to individuals without CIPN symptoms. Specifically, Kolb et al. [6] reported that of 116 individuals receiving neurotoxic chemotherapy, individuals with sensory CIPN symptoms (*n* = 32) were 2.7 times more likely to have a fall or near fall event than those without sensory CIPN symptoms. Decreased physical function due to unmanaged CIPN symptoms may result in 1) increased health care utilization (e.g., cost of care) [10], 2) the inability to return to work immediately following treatment [11], and 3) difficulty completing daily activities [12]. However, despite the known negative effects of unmanaged CIPN symptoms on physical function, CIPN is not routinely assessed in clinical practice [13, 14]. Novel interventions are needed that prioritize the early identification of CIPN symptoms to provide prompt treatment (e.g., chemotherapy dose modification or supportive care strategies such as duloxetine) [15, 16] and referral to rehabilitation services [17–19] to reduce the impact of unmanaged CIPN symptoms on physical function [9].

Electronic care planning systems that 1) facilitate patient-clinician discussions about cancer treatment-related toxicities, 2) provide recommendations for self-care and referral to other support services, and 3) promote patients’ activation in cancer symptom management have shown promise in improving the assessment and management of cancer-related symptoms, such as CIPN, in clinical practice [20–23]. The Electronic Symptom Assessment – Cancer (ESRA-C) [23] is a web-based symptom self-report and self-care intervention that collects patient reported data through the administration of standardized questionnaires in between and prior to the patients’ clinic visits. In addition, based on the patient’s symptoms and/or quality of life issues, the platform provides patients with self-care education, instructions about how to communicate symptoms to the provider, and the ability to monitor symptom progression over time (e.g., graphs, journal). Further, the platform creates a summary of reports for clinicians about symptoms (i.e., based on severity scores associated with patient reported outcome measures). The use of these embedded tools may facilitate the early identification and management of CIPN symptoms via increased patient self-report and patient-provider communication about CIPN symptoms. Despite inclusion of physical function and CIPN assessment and self-care strategies, a formal comparison of the benefits of the ESRA-C have not been reported for patients receiving platinum or taxane-based chemotherapy.

### Purpose

The purpose of this analysis is to compare self-reported physical function of participants receiving platinum and taxane-based chemotherapy regimens randomized to the ESRA-C intervention or electronic symptom assessment alone. Secondary measures include pain intensity, sensory CIPN, motor CIPN, fatigue, depression, and insomnia.

## Methods

### Subjects

Subjects included in this analysis are a subset of those who participated in a multicenter, randomized-controlled trial [23]. Participants are included if they received a platinum (i.e., oxaliplatin or cisplatin) or taxane-based (i.e., paclitaxel or docetaxel) chemotherapy regimen and completed the baseline (T1) and end-of-treatment (T3/4) time points. Participants were recruited from two large comprehensive cancer centers in Seattle, WA and Boston, MA. All data in this analysis came from participants receiving care in Boston because Seattle participation was limited to bone marrow transplant recipients.

### Measures

#### European organisation for research and treatment of cancer quality of life core questionnaire 30 (EORTC QLQ C-30)

The QLQ C-30 is a 30-item measure that consists of 15 functional and/or symptom scales [24]. To evaluate the impact of neurotoxic chemotherapy on physical function, we used the five-item Physical Functioning Scale. The Physical Functioning Scale measures patient’s perceptions of difficulty with activities of daily living, strenuous activity, and walking over the past week. Each of the five items is rated on an 1–4 scale. The total score is transformed to a 0–100 scale, with higher scores representing greater physical function [25]. The QLQ C-30 Physical Functioning Scale was used as the primary measure of physical function in this analysis (physical functioning impairments due to CIPN were measured separately) because the scale is the most commonly administered measure of physical function in cancer clinical trials [26] and a myriad of co-occurring symptoms (e.g., anxiety, depression, fatigue, pain, insomnia) related to chemotherapy administration may lead to reductions in physical function [27–29], not CIPN alone. The 3-item fatigue and 1-item insomnia symptom scales were used to measure the secondary measures of fatigue and insomnia, respectively. The Fatigue Symptom Scale measures self-reported feelings of tiredness (e.g., need for rest) during the day over the past week. The Insomnia Symptom Scale asks participants if they had trouble sleeping during the night over the past week. The items of each respective measure are scored on an 1–4 scale, with total transformed scores ranging from 0 to 100 (higher scores represent worse severity) [25]. Several studies provide evidence supporting the reliability and validity of the QLQ C-30 for use in individuals with cancer [24, 30, 31].

#### EORTC QLQ CIPN20

The QLQ-CIPN20 is comprised of three subscales that measure CIPN symptoms and associated functional deficits specific to each subscale [32]. Only responses to the sensory and motor subscales were evaluated in this study because recent studies indicate that the autonomic subscale has suboptimal reliability and/or validity [33]. The items of the sensory and motor subscales are scored on an 1–4 scale, with total transformed scores ranging from 0 to 100 (higher scores represent worse neuropathy). Studies have reported high reliability, discriminant validity, and responsiveness to change of the sensory and motor subscales [33]. To further explore the effect of the ESRA-C intervention on sensory and motor CIPN, we categorized participants based on the amount of neurotoxic chemotherapy received during the study. The categories (low, moderate, or high dose) were created based on cumulative dose ranges associated with increased CIPN severity [34–37]. The footnote of Table 1 further describes the dose ranges for paclitaxel, docetaxel, cisplatin, and oxaliplatin that constituted the low, moderate, and high dose categories.

#### Patient health questionnaire 9 (PHQ-9)

The PHQ-9 measures patient’s perceptions of their mood (e.g., feeling tired, lack of interest in doing things) over the past two weeks. Each item is scored from 0 to 3, with total scores ranging from 0 to 27 (higher scores represent worse depression) [38]. The PHQ-9 has demonstrated sufficient internal consistency reliability [39] and construct validity (strong associations between PHQ-9 scores and functional status) [39].

#### 0–10 current pain intensity numerical scale (PINS)

Participants rated current pain on a scale of 0–10, with a score of “0” representing “no pain,” and a score of “10” representing the “worst pain you can imagine.” The use of a 0–10 numerical rating scale of pain intensity is recommended by the Initiative on Methods, Measurement, and Pain Assessment in Clinical Trials [40] and the scale’s strong psychometric properties are well documented [41, 42].

### Procedures

The primary randomized-controlled trial was fully reported elsewhere [23]. Briefly, following informed consent, participants completed the baseline assessments (e.g., QLQ-C30, QLQ-CIPN20, PHQ-9) via home computer or computer tablet before beginning their new chemotherapy regimen (T1). Participants were then randomized (via computer application) in an 1:1 ratio to the intervention or control group (blocks of four based on self-identification as a home or clinic user). Participants in both groups used the ESRA-C program to self-report symptoms and quality of life measures at each clinic visit and the intervention group could access the program at home (at their discretion) in between clinic visits. When an intervention group participant reported a specific symptom as moderate to severe (see Table 3 for cut points associated with each measure), the software prompted the participants to view three self-care messages: 1) education about why the symptom occurs, 2) how the symptom can be managed (e.g., links to local content and from national cancer organizations), and 3) how to talk to providers about the symptom. Intervention group participants also could use the ESRA-C to track symptom patterns at home by viewing graphs that displayed symptom severity scores over time and documenting self-care activities in a journal. CIPN-related self-care information focused on how to stay safe with neuropathy symptoms (e.g., wear gloves when outside in cold temperatures, using hot water, or handling sharp objects; use non-slip rugs in the house, ensure house is well lit when walking at night) [19]. Intervention group participants could access all self-care messages at any time during the study regardless if they scored above the pre-defined symptom threshold. Control group participants did not receive self-care messages for problematic symptoms or have the ability to track symptoms over time. Within 24 h of each clinic visit, the ESRA-C was to be completed, and participants in both groups received usual education (e.g., clinician assessment and symptom management education) and the clinicians received a graphical summary of symptom severity scores. All participants completed the assessment measures via the ESRA-C three to six weeks after beginning a new chemotherapy regimen (T2), two four weeks after the second-time point (T3), and two to four weeks after treatment completion (T4). If a participant finished treatment before T3, the T4 assessment was not administered. Information pertaining to enrolled participants’ cancer diagnosis, neurotoxic chemotherapy type, and neurotoxic chemotherapy dose was abstracted from the electronic medical record during the study.

### Statistical analysis

Descriptive statistics are calculated for all measures at each time point. Boxplots are used to graphically show the changes over time. Due to the low amount of missing data for the primary and secondary outcomes (< 5%), mean imputation is used to handle missing data. Baseline characteristics are compared using Wilcoxon rank-sum test and Fisher’s exact test for continuous and categorical variables, respectively.

In the analysis, a mixed effects model allowing for subject specific intercept and slopes is used to evaluate if there are significant differences in the changes in physical function, fatigue, insomnia, depression, sensory CIPN, motor CIPN, and pain intensity scores over time between the two groups. This model adjusted for age at baseline (T1). All data are analyzed using R version 3.3.2 [43]. The *lme* function of the *nlme* package of the R language is used for linear mixed models [44]. Results with *p*-values ≤0.05 are considered statistically significant. No adjustment for multiple outcome comparisons is performed for the secondary measures. The interpretation of the time by treatment interaction coefficient from the mixed model depend on the direction of the time effect. For example, if the scores are decreasing over time and the interaction coefficient is negative (positive), this means the intervention group scores decrease at a higher (lower) rate than the control group. Alternatively, if the scores are increasing over time and the interaction coefficient is negative (positive), this means the intervention group scores increase at a lower (higher) rate than the control group scores.

To provide information on how often intervention group participants were prompted to view the ESRA-C self-care messages, we report the frequency of intervention group participants’ symptom severity scores that were high enough to prompt the receipt of the self-care messages.

## Results

### Sample characteristics

Of the 752 (374 intervention; 378 control) enrolled participants in the primary, randomized-controlled trial, 220 (29.2%) (108 intervention; 112 control) are eligible for this analysis. We initially excluded 482 participants from this analysis because these participants were not receiving neurotoxic chemotherapy (i.e., oxaliplatin, docetaxel, paclitaxel, or cisplatin). Next, of the 270 (140 intervention; 130 control) participants receiving neurotoxic chemotherapy, 50 participants were excluded from this analysis because they did not complete the T3/4 outcome assessments (i.e., 42% lost to follow up, 36% voluntary withdrawal, 22% other reasons such as death). The subjects at T1 were a median age of 54 (*Range* = 22–87) years old, mainly female, Caucasian, and employed/working. In terms of cancer treatment characteristics, most subjects: 1) received care in the medical oncology service, 2) had a diagnosis of breast cancer, 3) received taxane-based chemotherapy, and 4) had Stage III or IV cancer severity. There were no statistically significant between-group differences in demographic or cancer treatment-related characteristics at baseline (Table 1, *p* > 0.05). Baseline scores for the primary or secondary outcomes were not significantly different by treatment group for any of the measures (Table 2, *p* > 0.05 via two-sample t-test).

**Table 1:** Demographic and Cancer Treatment-Related Characteristics (*N* = 220)

Variable	Intervention (*N* = 108)	Control (*N* = 112)	*p* value
Age at Baseline			*p* = 0.33
Median (*Range*)	54 (26–86)	56 (22–87)	
Time on Study (Days)			*p* = 0.64
Median (*Range*)	122 (49–482)	113 (49–378)	
Gender			*p* = 0.89
Male	51 (47%)	51 (46%)	
Female	57 (53%)	61 (54%)	
Ethnicity/Race			*p* = 0.95
Caucasian	94 (87%)	98 (87%)	
Minority	4 (4%)	3 (3%)	
Missing	10 (9%)	11 (10%)	
Working Status			*p* = 0.49
Working	72 (67%)	79 (71%)	
Not working	29 (27%)	23 (20%)	
Missing	7 (6%)	10 (9%)	
Clinical Service			*p* = 0.83
Medical Oncology	95 (88%)	100 (89%)	
Radiation Oncology	13 (12%)	12 (11%)	
Cancer Diagnosis^a^			*p* = 0.83
Breast	34 (31%)	42 (38%)	
Head and Neck	17 (16%)	14 (12%)	
Colorectal	16 (15%)	14 (12%)	
Prostate	12 (11%)	10 (9%)	
Esophageal	7 (6%)	3 (3%)	
Testicular	7 (6%)	5 (4%)	
Gastrointestinal Other	4 (4%)	2 (2%)	
Other	4 (4%)	3 (3%)	
Sarcoma	3 (3%)	0	
Bladder	2 (2%)	9 (8%)	
Gastric	2 (2%)	5 (4%)	
Pancreatic	0	1 (1%)	
Unknown Primary	0	4 (4%)	
Stage			*p* = 0.29
I	14 (13%)	19 (16%)	
II	30 (28%)	29 (26%)	
III	30 (28%)	22 (20%)	
IV	30 (28%)	40 (36%)	
Missing	4 (4%)	2 (2%)	
Chemotherapy Type			*p* = 0.98
Taxane Only	62 (58%)	62 (55%)	
Platinum Only	39 (36%)	42 (38%)	
Platinum and Taxane	7 (6%)	8 (7%)	
Cumulative Dose Category^b^			*p* = 0.31
Low Dose^c^	25 (23%)	26 (23%)	
Moderate Dose^d^	35 (32%)	48 (43%)	
High Dose^e^	47 (44%)	37 (33%)	
Missing	1 (1%)	1 (%)	
Neurotoxic Chemotherapy Dose Reduction			*p* = 0.60
None	96 (89%)	104 (93%)	
Neuropathy-Related	9 (8%)	6 (5%)	
Other Symptom-Related^f^	3 (3%)	2 (2%)	

This table describes the demographic characteristics of the patients at baseline

^a^To compare distribution of cancer diagnosis for intervention versus control, the diagnoses with < 10 observations are grouped together into another category (i.e., esophageal, testicular, gastrointestinal, miscellaneous, sarcoma, bladder, gastric, pancreatic and unknown primary)

^b^For participants receiving multiple neurotoxic chemotherapy agents, dose category was determined based on the highest dose of one of the specific agents they were receiving

^c^Paclitaxel < 700 mg/m^2^; Oxaliplatin < 800 mg/m^2^; Docetaxel < 300 mg/m^2^; Cisplatin < 300 mg/m^2^

^d^Paclitaxel 700–1400 mg/m^2^; Oxaliplatin 800–1000 mg/m^2^; Docetaxel 300–600 mg/m^2^; Cisplatin 300–600 mg/m^2^

^e^Paclitaxel > 1400 mg/m^2^; Oxaliplatin > 1000 mg/m^2^; Docetaxel > 600 mg/m^2^; Cisplatin > 600 mg/m^2^

^f^Neurotoxic chemotherapy dose reduction due to other symptom-related causes included fatigue, pain, skin changes, bowel problems, or breathing problems

**Table 2:** Scores for Primary and Secondary Outcomes Over Time (*N* = 220)

	Mean (*SD*)		Mean Change from Baseline (*SD*)		Model Estimate (*95% CI*, *p*)^b^
Outcomes^a^	Intervention	Control	Intervention	Control	Interaction effect
Physical Function					
Baseline	87.4 (15.6)	90.2 (12.7)			2.75 (0.65, 4.86), *p* = 0.011
Time 2	85.2 (17.5)	83.9 (16.7)	−2.2 (15.2)	−6.3 (14.1)	
Time 3/4	84.5 (16.1)	81.8 (17.0)	−2.9 (15.0)	−8.5 (16.8)	
Depression					
Baseline	4.2 (4.4)	3.5 (3.4)			−0.72 (−1.23, − 0.22) *p* = 0.005
Time 2	4.9 (4.0)	5.4 (3.3)	0.7 (3.5)	1.9 (3.2)	
Time 3/4	3.9 (3.6)	4.64 (4.3)	−0.3 (3.4)	1.2 (4.2)	
Fatigue					
Baseline	27.7 (20.5)	23.8 (18.9)			− 3.12 (−6.29, 0.04) *p* = 0.053
Time 2	37.6 (23.5)	41.0 (22.8)	9.8 (21.0)	17.3 (22.7)	
Time 3/4	34.4 (21.8)	36.7 (21.0)	6.7 (21.4)	12.9 (23.8)	
CIPN Sensory					
Baseline	3.9 (9.6)	3.3 (6.6)			−1.67 (−3.90, 0.55) *p* = 0.141
Time 2	7.8 (11.9)	6.4 (10.3)	4.0 (9.7)	3.1 (9.1)	
Time 3/4	12.9 (14.3)	15.7 (18.2)	9.0 (14.2)	12.4 (19.0)	
CIPN Motor					
Baseline	4.5 (10.1)	2.5 (4.0)			−1.3 (−2.95, 0.35) *p* = 0.122
Time 2	5.7 (11.5)	5.3 (7.4)	1.2 (6.2)	2.8 (6.6)	
Time 3/4	8.9 (12.7)	9.6 (13.4)	4.5 (11.7)	7.1 (13.1)	
Current Pain Intensity					
Baseline	1.7 (1.9)	1.5 (1.9)			−0.09 (−0.37, 0.18) *p* = 0.500
Time 2	1.72 (1.9)	1.6 (1.9)	0.03 (2.3)	0.10 (2.2)	
Time 3/4	1.7 (1.9)	1.7 (1.8)	0.00 (1.8)	0.19 (2.2)	
Insomnia					
Baseline	28.4 (24.9)	27.7 (26.8)			−0.62 (−4.58, 3.35) *p* = 0.760
Time 2	31.2 (29.6)	33.6 (25.9)	2.8 (31.6)	6 (28.7)	
Time 3/4	28.3 (24.9)	28.9 (27.0)	−0.05 (24.6)	1.19 (32.2)	
High Cumulative Dose Category (*n* = 84)					
CIPN Sensory					
Baseline	4.0 (12.3)	3.5 (6.2)			−5.70 (−9.89, −1.60) *p* = 0.007
Time 2	8.1(12.7)	7.7 (13.0)	4.1 (10.8)	4.2 (12.7)	
Time 3/4	13.8 (14.3)	24.7 (22.4)	9.8 (14.0)	21.2 (23.9)	
CIPN Motor					
Baseline	4.2 (10.7)	2.0 (3.4)			−3.45 (−6.70, −0.19) *p* = 0.039
Time 2	4.9 (10.3)	5.1 (7.3)	0.7 (6.0)	3.2 (6.9)	
Time 3/4	8.6 (10.7)	13.3 (17.8)	4.4 (12.9)	11.3 (17.6)	

This table describes differences in mean scores for the primary and secondary outcomes over time between treatment groups. There were no statistically significant differences in baseline scores between groups for any outcome (*p* > 0.05)

^a^Outcomes include the mean and standard deviation at each time point, the mean change from baseline and standard deviation of this change at the two follow-up time points, and the results from fitting a mixed model

^b^This effect represents interaction of time and treatment. For example, if the scores are decreasing over time and the interaction coefficient is negative (positive), this means the intervention group scores decrease at a higher (lower) rate than the control group scores. Alternatively, if the scores are increasing over time and the interaction coefficient is negative (positive), this means the intervention group scores increase at a lower (higher) rate than the control group scores. In this data, scores for all outcomes except for the physical assessment are increasing over time (main effects) and interaction coefficients are negative. Therefore, the interpretation for all outcomes except for physical function is that the intervention group scores increase at a lower rate than the control group scores. For physical function, the intervention group scores decrease at a lower rate as compared to the control group scores

### Physical function

**Fig. 1:**
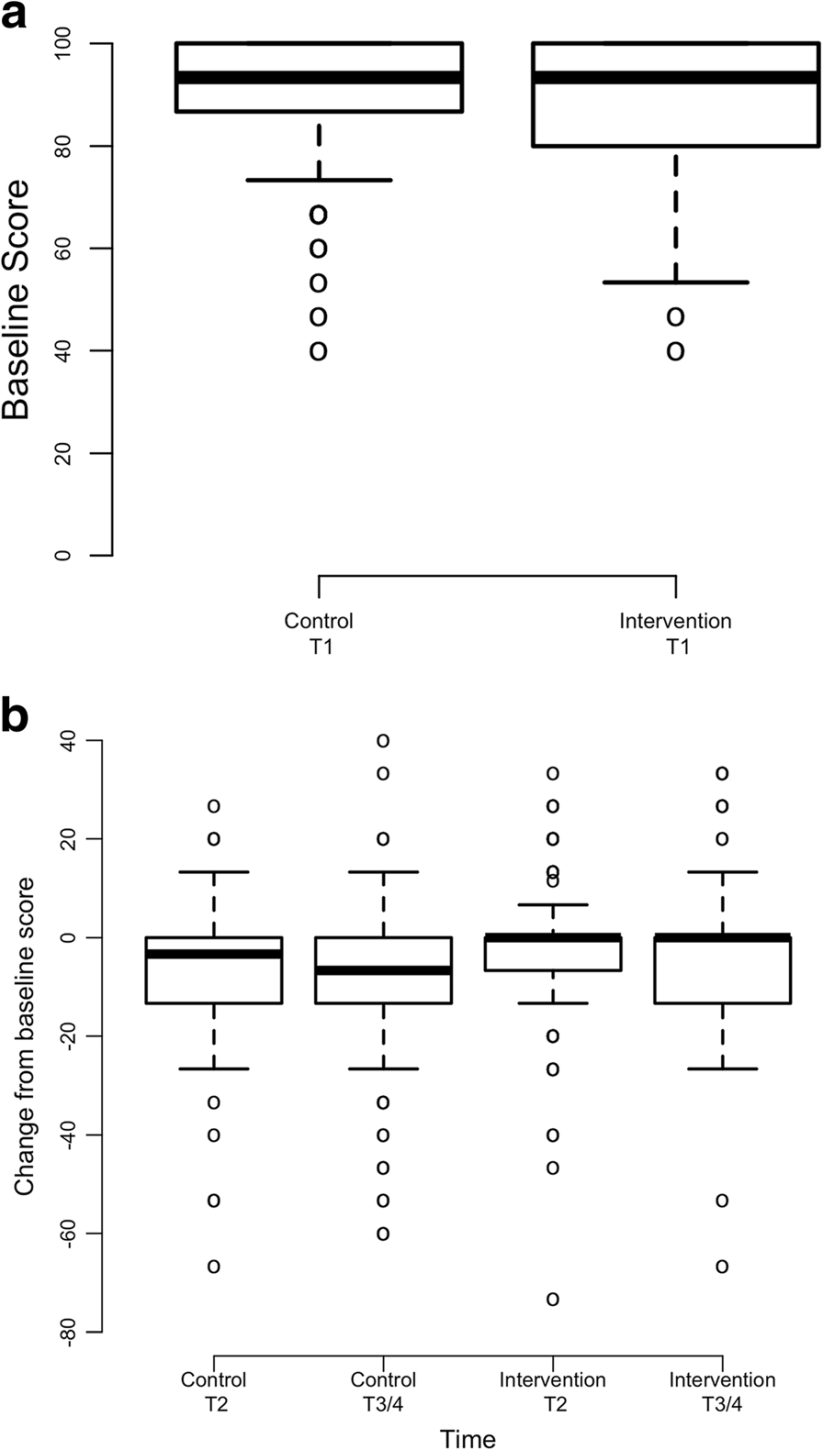
Physical Function Scores Across Time by Treatment (*N* = 220). **a** Baseline Physical Function Scores; Note: Higher scores represent greater physical function at baseline. **b** Mean Change in T2 and End of Study (T3/4) Physical Function Scores from Baseline. Note: Negative scores represent decreased physical function from baseline

Intervention group participants reported decreases in QLQ-C30 Physical Functioning Scale scores from 87.4 (*SD* = 15.6) at baseline to 84.5 (*SD* = 16.1) at T3/4, whereas control group participants reported decreases in QLQ-C30 Physical Functioning Scale scores from 90.2 (*SD* = 12.7) at baseline to 81.8 (*SD* = 17.0) at T3/4 (Fig. 1). The difference in physical function between groups over time was statistically significant (interaction coefficient = 2.75, *p* = 0.011) (Table 2). Since the physical scores are decreasing over time and the interaction coefficient is positive, the intervention group scores decrease at a lower rate than the control group.

### Secondary measures

**Fig. 2:**
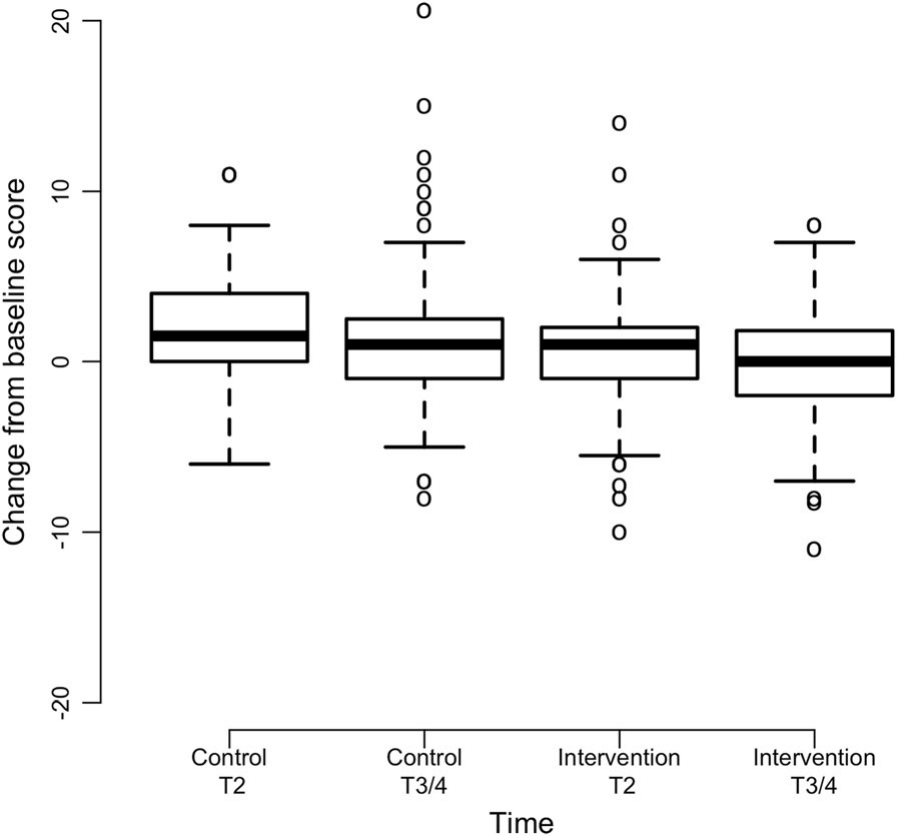
Mean Change in T2 and End of Study (T3/4) Depression Scores from Baseline (*N* = 220). Note: Positive scores represent increased depression severity from baseline

**Fig. 3:**
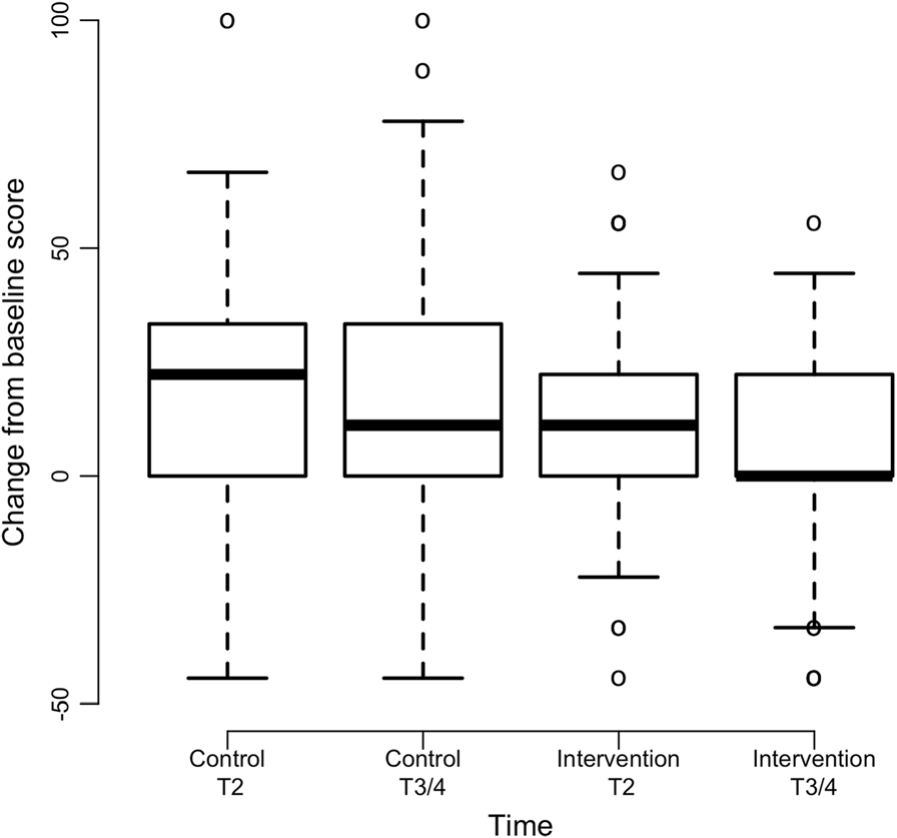
Mean Change in T2 and End of Study (T3/4) Fatigue Scores from Baseline (*N* = 220). Note: Positive scores represent increased fatigue severity from baseline

**Fig. 4:**
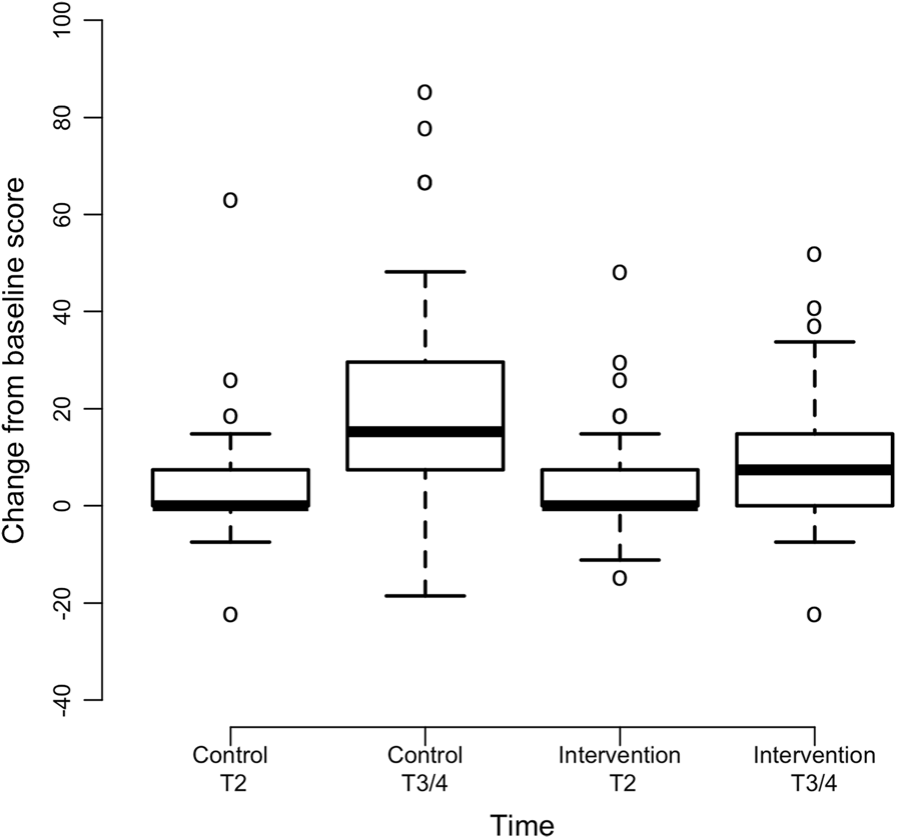
Mean Change in T2 and End of Study (T3/4) Sensory CIPN Scores from Baseline in Participants Receiving High Cumulative Neurotoxic Chemotherapy Dosages (*N* = 84). Note: Positive scores represent increased sensory CIPN severity from baseline

**Fig. 5:**
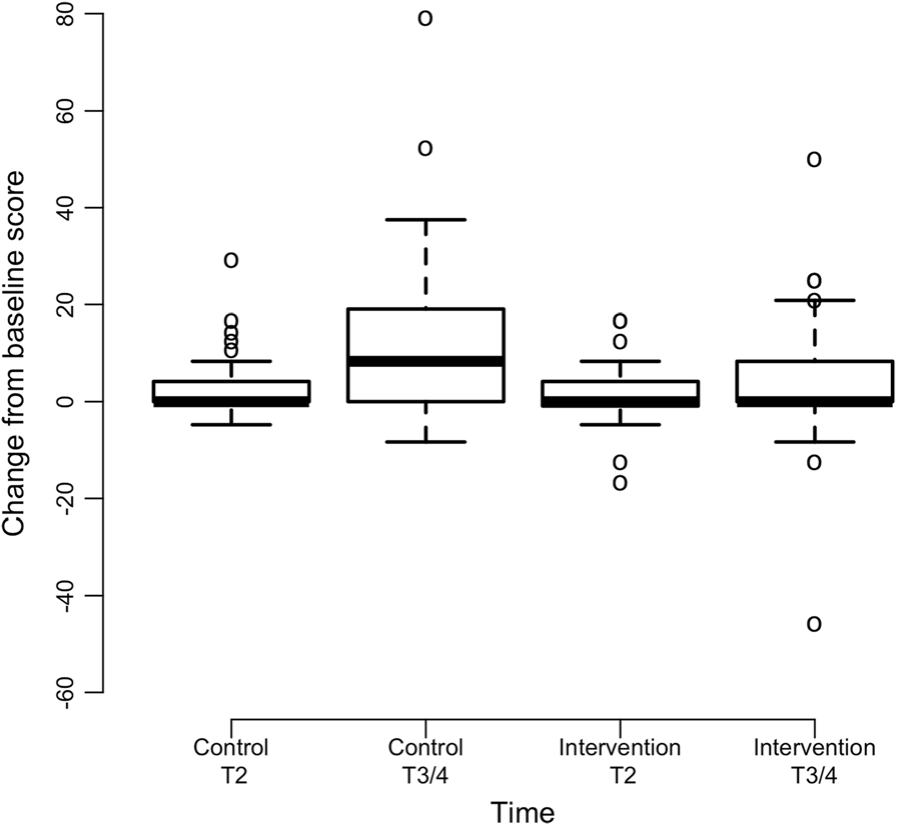
Mean Change in T2 and End of Study (T3/4) Motor CIPN Scores from Baseline in Participants Receiving High Cumulative Neurotoxic Chemotherapy Dosages (*N* = 84). Note: Positive scores represent increased motor CIPN severity from baseline

Intervention group participants experienced significantly less depression in comparison to control group participants (interaction coefficient = − 0.72, *p* = 0.005) (Fig. 2). Since the depression scores are higher at post baseline assessments relative to baseline and the interaction coefficient is negative, the intervention group scores increase at a lower rate than the control group. Differences in fatigue severity over time were marginally significant (*p* = 0.053) and in favor of the intervention group (Fig. 3). There were no significant differences in mean scores over time between groups for the variables of sensory CIPN (*p* = 0.141), motor CIPN (*p* = 0.122), pain intensity (*p* = 0.500), or insomnia (*p* = 0.760) (Table 2). Results from an exploratory subgroup analysis (*n* = 84) revealed that within individuals who were receiving high cumulative neurotoxic chemotherapy dosages [34–37], intervention group participants reported less severe sensory CIPN symptoms with maximum change of 9.8 as compared to 21.2 for control participants (*p* = 0.007) as well as less severe motor CIPN symptoms with maximum change of 4.4 as compared to 11.3 for control participants (*p* = 0.039) (Table 2; Figs. 4 and 5).

### Prompted ESRA-C intervention messages

Table 3 describes the number of times that intervention group participants scored above the pre-specified severity threshold associated with each symptom questionnaire to prompt the receipt of symptom specific ESRA-C self-care messages. Overall, the frequency of prompted self-care messages across the three time points for physical function (*Range* = 2–5%), sensory CIPN (*Range* = 1–3%), motor CIPN (*Range =* 2–4%), and depression (*Range* = 9–14%) were low, while the frequency of prompted self-care messages for fatigue (*Range* = 28–44%), insomnia (*Range* = 17–31%), and current pain intensity (*Range* = 17–21%) were higher.

**Table 3:** Frequency of Intervention Group Participants Scoring Above the Pre-Specified Symptom Severity Thresholds to Prompt Receipt of ESRA-C Self-Care Messages *(N* = 108*)*

Outcome	Cut Point	T1	T2	T3/4
Physical Function	QLQ – C30 Physical Function Subscale ≤50	2 (2%)	5 (5%)	5 (5%)
Sensory CIPN	QLQ – CIPN20 Sensory Subscale ≥ 50	1 (1%)	2 (2%)	3 (3%)
Motor CIPN	QLQ – CIPN20 Motor Subscale ≥50	2 (2%)	3 (3%)	4 (4%)
Current Pain Intensity	Pain Intensity Numerical Rating Scale ≥5 and/or Symptom Distress Pain Intensity Item ≥ 3^a^	18 (17%)	23 (21%)	18 (17%)
Fatigue	Symptom Distress Scale Fatigue Item ≥ 3^a^	30 (28%)	48 (44%)	34 (32%)^b^
Insomnia	Symptom Distress Scale Insomnia Item ≥ 3^a^	23 (22%)^c^	33 (31%)	18 (17%)
Depression	Patient Health Questionnaire – 9 ≥ 10	15 (14%)	12 (11%)	10 (9%)

This table describes the number of intervention group participants who scored above the pre-specified threshold associated with each patient-reported outcome measure that prompted receipt of symptom specific ESRA-C self-care messages

^a^Symptom Distress Scale – 15 Items [23, 61] scored from 1 to 5, with higher scores representing worse symptoms

^b^*n* = 107

^c^*n* = 105

## Discussion

The findings from this secondary analysis revealed that intervention group participants reported less reduction in physical function in comparison to control group participants following use of the ESRA-C. Control group participants’, but not intervention group participants’, mean physical function score at the end of treatment met the threshold for clinical importance (≤ 83/100) [45] and mean change in physical function score from baseline to posttest approached the minimal clinically meaningful difference for deterioration ( -9) [46]. However, our results should be interpreted with caution as there were no differences in sensory or motor CIPN severity between groups. To our knowledge, this analysis is among the first to explore the efficacy of a web-based symptom assessment and management platform to preserve physical function in a large sample of individuals receiving neurotoxic chemotherapy. Previous studies have examined the implementation of electronic assessment and management platforms in practice on outcomes such as technology usability [20], patient activation [21], and provider documentation of CIPN assessment and management [47] in individuals receiving neurotoxic chemotherapy. Additionally, Tofthagen and colleagues developed an algorithm to guide nursing management of CIPN symptoms and associated functional impairment [48] that was later incorporated into a web-based psychoeducational intervention for individuals with acute CIPN symptoms. Results of the intervention study revealed that use of the web-based psychoeducational program led to improvements in pain-related interference (*d* = 0.39, *N* = 14) [49]. Finally, the authors of a secondary analysis evaluated an automated telephone symptom reporting system for chemotherapy-related neuropathic pain management in patients receiving taxane or platnium-based chemotherapy agents. During chemotherapy, intervention and control group participants (*N* = 252) called the telephone system every day to self-report chemotherapy-related symptoms, but intervention group participants received automated self-management information and nurse practitioner follow up phone calls for more severe symptoms. Participants in the intervention group reported less days with moderate (*p* < 0.001) and severe CIPN symptoms (*p* = 0.006) and decreased activity interference due to CIPN (*p* = 0.08) in comparison to control group participants [22]. 

While intervention group participants reported less reduction in physical function in comparison to control group participants, little is known about the possible mechanisms by which physical function was preserved. The principles of causal mediation state that the intervention must significantly affect the hypothesized mediator in comparison to the control for mediation to occur [50]. Depression was the only symptom where significant differences were observed between treatment groups. While it is possible that depression was a significant mediator of physical function preservation, it is unlikely that improvements in depression alone mediated physical function preservation as the change in mean depression severity score between groups was not clinically significant [51]. Further, our original hypothesis was that interventions like the ESRA-C may work to prevent reductions in physical function in individuals receiving neurotoxic chemotherapy by increasing the early identification and management of CIPN (e.g., referral to specialty services such as neurology or physical therapy, chemotherapy dose modification). As there were no formal clinical practice guidelines for CIPN management when the original trial was conducted (2009–2011), chemotherapy-dose reduction would have been one of the primary treatments for patients with CIPN [19]. Thus, our results provide evidence against our initial hypothesis as there were no differences in sensory or motor CIPN severity between treatment groups and there were no differences in the frequency of neurotoxic chemotherapy dose reduction due to CIPN. We may not have observed differences in many of the secondary outcomes because symptom severity scores were low, and few participants scored high enough on the associated measures to prompt the receipt of symptom specific self-care messages. As such, it is puzzling that in individuals receiving cumulative neurotoxic chemotherapy dosages that were associated with more severe CIPN, intervention group participants reported less severe sensory and motor CIPN than control group participants. A replication study in the future may be warranted as more is currently known about evidence-based treatments for CIPN (i.e., duloxetine 60 mg/day) [15, 16].

It is possible that intervention group participants reported better physical function at posttest in comparison to control group participants because they experienced small improvements in all symptoms. This hypothesis may be partially explained using the Theory of Unpleasant Symptoms (TOUS) as a guiding framework [52]. Per the TOUS, it is hypothesized that physical and psychological influencing factors (i.e., depression, insomnia, fatigue), symptoms (i.e., sensory/motor CIPN, pain intensity), and performance outcomes (i.e., physical function) all influence one another. Intervention group participants may have been able to experience small improvements in multiple symptoms because while few scored high enough on the study specific scales associated with each outcome measure to prompt the receipt of self-care messages, previous research suggests that a majority (233/374, 62.3%) of intervention group participants in the original trial were exposed to the ESRA-C intervention (e.g., clicked on self-care messages or symptom reports at least twice) [53]. Many intervention group participants may still have been able to use the strategies (e.g., patient-provider communication strategies) and self-care information to increase patient activation in cancer treatment-related symptom management [21]. In sum, our findings highlight the need for further research to determine variables that mediate physical function preservation following the implementation of web-based symptom assessment and management interventions to tailor such interventions in the future to variables known to positively influence physical function.

Due to the lack of differences in sensory or motor CIPN between groups, the ESRA-C intervention may be modified in the future to further bolster strategies for the self-management of CIPN and associated symptoms to ultimately preserve physical function. Recent evidence has revealed that increased BMI and low participation in moderate-vigorous physical activity are associated with the development of CIPN [54]. Thus, self-management programs that encourage daily physical activity may be a promising treatment approach. Two recent prospective trials provide evidence supporting the efficacy of exercise interventions in reducing CIPN symptoms and/or improving function in patients receiving chemotherapy [55, 56]. Further, cognitive behavioral pain management strategies (e.g., sleep hygiene, relaxation, activity pacing) [57] have been shown to have a positive effect on common cancer treatment-related symptoms (e.g., pain, anxiety, fatigue, insomnia) [58–60]. Specifically, the authors of a recent randomized controlled trial demonstrated that the delivery of cognitive behavioral pain management strategies via the internet may significantly improve worst CIPN pain severity in individuals with chronic painful CIPN [60]. Taken together, incorporating exercise and cognitive behavioral strategies into web-based symptom assessment and management platforms like the ESRA-C, may provide patients with additional efficacious self-management strategies to improve CIPN symptoms and preserve physical function during neurotoxic chemotherapy treatment.

The results of this study have several implications for practice. Nurses may consider implementing common components of web-based technology such as the ESRA-C to facilitate patient-nurse communication about cancer treatment-related symptoms during neurotoxic chemotherapy administration. Components of the ESRA-C that nurses may translate to their practice include: 1) using standardized patient-reported outcome measures to help patients identify cancer treatment-related symptoms and 2) providing self-care instruction for cancer treatment-related symptoms. Increased patient-nurse communication about cancer treatment-related symptoms may lead to earlier identification and management of symptoms, thereby preventing reductions in physical function in individuals receiving neurotoxic chemotherapy.

### Limitations

This study has several limitations. First, symptom severity scores for all secondary outcomes were fairly low, and therefore, we may not have observed any statistically significant differences between groups in some outcomes due to a floor effect. Similarly, there were many cases in which individuals in the intervention arm did not score high enough on the associated symptom measures to trigger the receipt of the additional ESRA-C self-care messages. This limitation is a potential threat to internal validity because in these cases, the content of the intervention and control arms were not significantly different. Second, the effect of the ESRA-C intervention on physical function was evaluated soon after the completion of chemotherapy treatment or late within the treatment course. It is important to evaluate long term outcomes in individuals with CIPN because CIPN symptoms may persist or worsen after the completion of treatment, especially following platinum-based chemotherapy [35]. Third, this study was conducted in a homogenous patient population (Caucasian, middle aged, receiving care at comprehensive cancer centers) and subsequently, our results may not be widely generalizable. Fourth, this analysis used a subset of the full sample from the primary study and may be underpowered for all outcome comparisons. Finally, patients and clinicians were not blinded to treatment assignment in the original randomized-controlled trial.

## Conclusion

CIPN is a common toxicity of neurotoxic chemotherapy treatment that can impair physical function during and months to years after the completion of treatment. The findings of this analysis suggest that use of a web-based symptom assessment and management platform (ESRA-C) may positively affect physical functioning during neurotoxic chemotherapy treatment. Yet, our results must be interpreted cautiously as this was an exploratory analysis, little is known as to how the ESRA-C intervention worked to prevent reductions in physical function, and there were no differences in sensory or motor CIPN severity between treatments. Overall, based on the positive results of this secondary analysis, and dearth of prospective studies, future research is needed to test interventions that improve the assessment and management of CIPN in clinical practice to prevent reductions in physical function related to unmanaged CIPN symptoms.

## Abbreviations

CIPN: Chemotherapy-induced peripheral neuropathy
EORTC QLQ C-30: European Organisation for Research and Treatment of Cancer Quality of Life Core Questionnaire 30
EORTC QLQ CIPN20: European Organisation for Research and Treatment of Cancer Quality of Life Chemotherapy-Induced Peripheral Neuropathy twenty-item scale
ESRA-C: Electronic Symptom Assessment – Cancer
PHQ-9: Patient Health Questionnaire 9
PINS: 0–10 Current Pain Intensity Numerical Scale
TOUS: Theory of Unpleasant Symptoms
