# Correction to: Exploring the efficacy of an electronic symptom assessment and self-care intervention to preserve physical function in individuals receiving neurotoxic chemotherapy

**DOI:** 10.1186/s12885-019-5669-2

**Published:** 2019-05-13

**Authors:** Robert Knoerl, Edie Weller, Barbara Halpenny, Donna Berry

**Affiliations:** 1 Phyllis F. Cantor Center for Research in Nursing and Patient Care Services, Dana-Farber Cancer Institute, 450 Brookline Avenue, LW 517, Boston, MA 02215 USA; 2 Biostatistics and Research Design Core, Institutional Centers for Clinical and Translational Research, Boston Children’s Hospital, 21 Autumn Street Suite 313, Boston, MA 02215 USA; 3 Phyllis F. Cantor Center for Research in Nursing and Patient Care Services, Dana-Farber Cancer Institute, 450 Brookline Avenue, LW 521, Boston, MA 02215 USA; 4 Phyllis F. Cantor Center for Research in Nursing and Patient Care Services, Dana-Farber Cancer Institute, 450 Brookline Avenue, LW 518, Boston, MA 02215 USA

**Correction to: Knoerl et al. BMC Cancer (2018) 18:1203**

**https://doi.org/10.1186/s12885-018-5093-z**

Following publication of the original article [1], the authors reported that the chemotherapy dosages in the footnote of Table 1 should be in mg, and not mg/m^2^.

The footnote for Table 1 should read as follows:

This table describes the demographic characteristics of the patients at baseline.

^a^To compare distribution of cancer diagnosis for intervention versus control, the diagnoses with < 10 observations are grouped together into another category (i.e., esophageal, testicular, gastrointestinal, miscellaneous, sarcoma, bladder, gastric, pancreatic and unknown primary).

^b^For participants receiving multiple neurotoxic chemotherapy agents, dose category was determined based on the highest dose of one of the specific agents they were receiving.

^c^Paclitaxel < 700 mg; Oxaliplatin < 800 mg; Docetaxel < 300 mg; Cisplatin < 300 mg.

^d^Paclitaxel 700–1400 mg; Oxaliplatin 800–1000 mg; Docetaxel 300–600 mg; Cisplatin 300–600 mg.

^e^Paclitaxel > 1400 mg; Oxaliplatin > 1000 mg; Docetaxel > 600 mg; Cisplatin > 600 mg.

^f^Neurotoxic chemotherapy dose reduction due to other symptom-related causes included fatigue, pain, skin changes, bowel problems, or breathing problems.
